# Dr. S. M. Burnett

**Published:** 1875-06

**Authors:** 


*Dr. S. M. Burnett.*

Our Associate, Dr. Burnett will, in a few weeks, leave his home, Knoxville, Tennessee, for Europe. While we much regret the temporary vacancy the Doctor will cause on our staff, for the time being, yet we feel assured his trip will ultimate in great good to science.—We shall miss his monthly contribution, his earnest zeal, and his kind and hearty co-operation ; yet we know he goes to enrich a mind already well stored with medical truths, and to return to enlarge a practice in a specialty he has already made a success.

Dr. Burnett will furnish our pages with letters from the great medical centres of the old world, which will form an attractive feature of the RECORD during his stay in Europe, and while we shall lose his presence, our freiends will be no losers by his absence abroad.

